# Longitudinal evaluation of a programme for safety culture change in a mental health service

**DOI:** 10.1111/jonm.13205

**Published:** 2020-11-22

**Authors:** Geoffrey L. Dickens, Yenna Salamonson, Alisha Johnson, Lucie Ramjan, Kelly Steel, Michelle Taylor, Bronwyn Everett

**Affiliations:** ^1^ Centre for Applied Nursing Research Ingham Institute for Applied Medical Research South Western Sydney Local Health District and Western Sydney University School of Nursing and Midwifery Liverpool NSW Australia; ^2^ School of Nursing and Midwifery Western Sydney University Penrith NSW Australia; ^3^ Taylor Made Coaching Solutions Sydney NSW Australia

**Keywords:** culture, mental health, nursing, organisational behaviour, patient safety, physiological deterioration

## Abstract

**Aim:**

To evaluate whether a two‐part culture improvement programme aimed at nurses in clinical and managerial positions in an inpatient mental health service was associated with culture change, and safety‐related behaviour and knowledge improvements.

**Background:**

Due to serious failings in the delivery of physiological care to mentally disordered inpatients, it was deemed important that interventions be applied to improve service culture.

**Methods:**

A pre‐test and post‐test study was conducted to evaluate change associated with a mandated intervention aimed at culture change. Nurses in clinical and managerial positions at all levels attended relevant sessions. All were invited to participate in evaluation measures.

**Results:**

*N* = 241 nurses participated in the evaluation (*n* = 137 and *n* = 104, pre‐test and post‐test, respectively). There was a small but significant change in organisational culture indicating greater *adhocracy* and less *clan* culture in the second survey period and a small decline in reported safety behaviour. Measures of safety culture, knowledge and emergency‐related educational satisfaction were unchanged.

**Conclusion:**

Only a small change in measured culture was associated with the programme.

**Implications for Nursing Management:**

Attempts to evaluate culture change need to align anticipated outcomes with appropriate outcome measures. A mandated programme of culture change had little tangible effect on the outcomes measured.

## 1 BACKGROUND

Failure to recognize and appropriately respond to clinical physiological deterioration in patients admitted to mental health units is a relevant factor in a significant proportion of adverse events within these settings (Findlay et al., 2012). There is considerable variability in the extent to which nurses in mental health settings engage in routine physical health care activity which might provide early warning signals of deterioration (Dickens et al., 2019) and in the availability of resources or training to support nurses to recognize and respond to severely clinically deteriorating patients in acute mental health settings (Dickens, et al., 2019; Sands et al., 2017). Importantly, resistant or negative attitudes from clinicians who regard their role as exclusively attending to patient's mental well‐being and who do not see the physical care of patients as part of their role, lack of organisational supports and leadership, poor team culture, distress and burnout, and reduced commitment to maintaining professional standards are also factors (Ward, 2005). Failings in physical health care in mental health settings can to some extent thus be understood in the context of prevalent organisational culture—typified by shared assumptions, shared ways of thinking and their visible manifestations (Mannion & Davies, 2018)—as much as in the context of expertise and technical skill.

The current study, conducted in Australia, was planned in the context of recommendations made in a 2017 New South Wales (NSW) government review of care in its mental health facilities (Wright 2017). Ms Miriam Merten, a recently detained inpatient at Lismore Base Hospital, NSW, died of a hypoxic brain injury caused by head injuries resulting from repeated falls. Nurses on duty either failed to recognize, or wilfully ignored, her deterioration (Ross, 2018) and the Coronial inquest noted that she ‘did not have her physical health assessed, particularly in regard to neuro observations’ (Coroners' Court of New South Wales, 2016). While disciplinary action was taken against the two nurses involved, the official reaction extended to an acknowledgement that Ms Merten's death represents issues of ‘culture and practice and leadership’ as much as individual culpability. The resulting review included 19 recommendations across six domains: culture and leadership, patient safety, accountability and governance, workforce, consumer engagement, and the built and therapeutic environment (Wright 2017). With particular reference to issues of culture and safety, the report recommended that NSW ‘must establish and adopt an integral leadership development framework’ and ‘adopt a mental health safety programme informed by contemporary improvement science’.

Given the issues raised by the incident described above, the official response for strategic action and the ongoing need for improvement in relation to organisational and safety culture in mental health inpatient settings, action was initiated at the district‐executive level in one local health district in Sydney, New South Wales. Namely, an independent, freelance organisational culture practitioner was contracted to deliver appropriate interventions aimed at supporting culture change: supporting nurse leaders and nurses to reframe and adapt existing cultural norms, goals, leadership styles, values and motivations, their approaches to change and problem solving and their attitudes within the context of changes to clinical practices such as patient observation, assessment and escalation of clinical deterioration. The purpose of the current study was to evaluate the impact of the culture‐change programme for nurses across a range of outcomes.

## 2 METHODS

### 2.1 Setting and participants

The study was conducted across the inpatient mental health services (11 wards at three hospital sites) of one metropolitan local health district in NSW, Australia. Eligible participants were registered nursing staff employed in direct clinical, educational or managerial/ senior managerial roles (*N* = 297) in the services at either data collection point.

### 2.2 Design

A longitudinal pre‐test and post‐test design was utilized. Baseline measures were taken, a service‐mandated programme of culture change was implemented, and follow‐up data gathered 1 year later.

### 2.3 Measures

#### 2.3.1 Safety attitudes questionnaire short form (SAQ‐SF; Sexton, Helmreich, Neilands, et al. 2006)

The SAQ‐SF is a 36‐item tool measuring the perceived extent of seven patient safety culture dimensions (see Table 1). Response is on a five‐point Likert scale. Factor structure has been supported by confirmatory factor analysis (Sexton et al., 2006), and internal reliability ranging from α = 0.742–0.897 for each scale has been reported (Dickens, et al., 2019).

**Table 1:** Sample characteristics at baseline and follow‐up

	Baseline (*n* = 137) *n* (%)	Follow‐up (*n* = 104) *n* (%)
Job role		
Clinician	121 (88.3)	91 (89.2)
Manager	14 (10.2)	0 (0.0)
Educator	0 (0.0)	1 (1.0)
Other	2 (1.5)	10 (9.8)
DNR	0 (0.0)	2 (2.0)
Employment status		
FT	111 (81.0)	92 (89.3)
PT	26 (19.0)	11 (10.7)
Education		
≤BN	96 (70.1)	80 (79.2)
BN+	10 (7.3)	5 (5.0)
M	29 (21.2)	15 (14.9)
Other	2 (1.5)	1 (1.0)
DNR	0 (0.0)	3 (2.7)
Hospital		
Site 1	27 (20.0)	17 (16.7)
Site 2	54 (40.0)	36 (35.3)
Site 3	54 (40.0)	49 (48.0)
Other	0 (0.0)	1 (1.4)
Years qualified	2.75 (1.08)	2.59 (0.99)
Years in MH	2.45 (1.04)	2.48 (1.01)
Years working in MH	2.20 (1.06)	2.34 (1.03)

#### 2.3.2 Safety behaviour (Elsous, Akbarisari, Rashidian, Aljeesh, Radwan, & Abu Zaydeh 2017)

This is a 3‐item scale which has previously been appended to the SAQ‐SF in a Palestinian study (Elsous et al., 2017). Response is on a 5‐point Likert scale. Internal reliability in a previous study in the current setting fell slightly short of acceptability (α = 0.691; Dickens, Salamonson, et al., 2019).

#### 2.3.3 Organisational climate: The organizational culture assessment instrument (OCAI)

**Figure 1:**
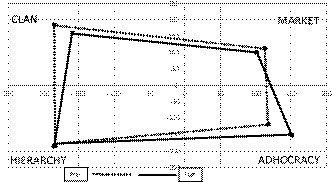
OCAI subscale scores pre‐ and post‐ culture programme

The OCAI is 24‐item scale premised upon the competing values framework (Cameron & Quinn, 2005) and measures features of shared organisational culture Cameron and Quinn, 1999. The conceptual framework posits two perceived dimensions, each representing competing values which are key to organisational culture: dimension (a) internal versus external focus of the organisation; and (b) organisational flexibility versus stability and control. Unique combinations of values (see Figure 1) represent organisational culture types. Hierarchy culture (internal focus, high stability and control) is typical for an organisation in which employees are process‐oriented. Clan culture (internal focus, high flexibility) represents a friendly and collaborative workplace. Adhocracy culture (external focus, high flexibility) represents a dynamic workplace with an entrepreneurial and creative environment. Market culture (external focus, high stability and control) describes an environment where employees are results‐oriented and competitive. OCAI items are presented in six blocks of four statements each of which reflect one organisational culture type. A ‘forced choice’ (ipsative) scoring system is used in which respondents allocate 100 points among each of the four statements in each block. Subscale scores, reflecting each organisational culture type, are derived by summing the scores allocated to each across the six blocks (possible range per subscale 0–600 per domain).

#### 2.3.4 Lambeth triage in situ questionnaire (LTIQ) (Lavelle, Attoe, Tritschler, & Cross 2017)

The LTIQ was designed for a UK study which evaluated simulation‐based physical health care skills training (Lavelle et al., 2017). Knowledge about medical deterioration is measured by appropriate response to vignettes describing medical emergency scenarios in relation to symptom recognition, escalation, planning and treatment, inter‐professional communication and handover, incident reporting, staff debriefing, local policy and procedures. Our amended version of the tool comprised 27 items relating to five vignettes, each requiring identification of between four and seven correct steps. Confidence in providing physical health care was measured on 6‐point Likert scales related to knowing policies and procedures, working effectively as a team, understanding the roles and responsibilities of individuals required, communicating effectively, collaborating with people from other professions and managing the situation overall.

#### 2.3.5 Medical emergency team (MET) survey

This was based on a locally developed survey tool devised for a previous educational needs analysis of nursing staff in relation to ‘Between the Flags’, a state‐wide Australian programme for improving recognition and response to deteriorating patients (http://www.cec.health.nsw.gov.au/patient‐safety‐programs/adult‐patient‐safety/between‐the‐flags). The questionnaire covered issues relating to attendance at, and perceived effectiveness of, various local educational sessions including advanced life support and sepsis, recent involvement in a MET call and the perceived importance of receiving training on ten aspects of MET‐related activity (sample items: ‘MET calling criteria’, ‘communication and leadership in a clinical emergency’). Each requires a response on a 5‐point Likert scale ranging from 1 (‘not at all important’) to 5 (‘very important’). This tool is available from the authors upon request.

#### 2.3.6 Intervention feasibility: Open‐ended item

A single open‐ended question allowing participants to add any additional comments about the study intervention was also included in the second iteration of the questionnaire.

### 2.4 Procedure

The study was approved by South Western Sydney Local Health District Human Research Ethics Committee (HE17/198) and was registered (ACTRN12618000191291) on the Australian and New Zealand Clinical Trial Registry. The study described here was conducted to evaluate any changes in selected study outcome measures associated with the mandated intervention. The intervention (see 2.5) was selected by a district‐level executive of the local health district and was implemented as a service development in which all nursing staff working in the mental health inpatient service were mandated to participate. Measures undertaken as part of the intervention were not available to the research team. For the research element of the study, participation was subject to informed consent and participation was voluntary. Baseline data were collected between March and September 2018 and follow‐up data between March and September 2019. On both occasions, study researchers distributed questionnaire packs. Responses were anonymous; however, to assist in pairing of pre‐test and post‐test data for analyses, respondents were requested to enter a code on the form comprising the first five letters of their mother's maiden name and the last three digits of their current telephone number on both occasions. The culture‐change interventions were implemented between May and October 2018.

### 2.5 Intervention

Interventions were planned and delivered by author MT an experienced organisational culture practitioner (Master of Applied Science). The overall objectives were to develop effective workplace cultures and nursing leadership within the selected units in order to (a) ensure the delivery of compassionate, safe, patient‐centred care through early recognition of and response to clinical deterioration; and (b) build on the work undertaken as part of a programme running concurrently in the study setting based on work developed by the NHS Institute for Innovation and Improvement (see e.g., WIlson, 2009) and aimed at releasing nursing staff time to engage with patients (the ‘productive mental health ward’). The focus for the culture interventions was on recognizing, facilitating and sustaining high‐performing positive team cultures. There were two culture‐change interventions: a nurse leader culture programme and a workplace culture programme. However, both used a similar range of approaches with the exception of psychometric assessment and individual coaching in the nurse leader programme (see 2.5.1). The programmes drew on multiple models and were not at all didactic. A coaching psychology approach incorporating a Socratic questioning approach informed by a 4‐D (discovery, dream, design and destiny) appreciative inquiry change methodology (Stavros & Hinrichs 2009) formed the backbone of the intervention. Programmes were also informed by theories of constructive conflict, strengths‐based approaches and active constructive responding (Seligman 2011); psychological safety and organisational learning (Edmondson 2019); and leadership skills (Ibarra 2015). The approach taken was to uncover participants' understanding of what was unique about the culture in each site and then discover how this compared with the desired culture.

#### 2.5.1 Nurse leader culture programme

This intervention was targeted at nurse leaders in the mental health services including the service‐level executive leadership, nurse managers and nurse unit managers. Key elements included (a) electronic administration of Hogan online personality assessments (Hogan Personality Inventory, Hogan Development Survey and Motives, Values Preferences Inventory [Hogan & Hogan, 2007]) and an individual, in person debriefing session lasting 1.5–2‐hr; (b) a leadership development programme comprising five 4‐hr sessions attended by the service‐level executive nursing team; (c) leadership development programme attended by nurse unit managers also comprising five 4‐hr sessions. The sessions were supplemented by interventions and activities facilitated by nurse leaders and champions at each study site.

#### 2.5.2 Workplace culture programme

This intervention was targeted at all ward nursing staff who were expected to attend three x 3‐hr sessions. To facilitate attendance, the sessions were run in multiple separate streams across three sites. Attendance registers for the workplace culture programme showed that 61.6% (183/297) of staff attended session one, 59.6% (177/297) attended session two, and 49.5% (147/297) attended session three. In total, 263/297 (88.6%) attended at least one session, 197/297 (66.3%) attended two or more sessions, and 91/297 (30.6%) attended all three sessions as mandated.

### 2.6 Data analysis

Data were entered into SPSS version 25.0 for analysis. Missing data were analysed at the outcome variable level with cases missing 10%+ of data for a variable excluded from the analysis listwise. Following listwise deletion of cases, there were 0.38% missing data points which were handled through multiple imputation. Inspection of participant‐generated anonymizing codes revealed that 17 individuals, termed here ‘repeaters’, had completed the questionnaires at both iterations (i.e. 12.4% of those taking the initial iteration had also to our certain knowledge repeated completion post‐intervention). For the remaining individuals, termed ‘non‐repeaters’, there was no evidence that they had completed both iterations since the anonymizing codes were either absent or were present on only one occasion. Because of the low proportion of repeat data we treated pre‐ and post‐intervention iterations as independent samples. Data distribution was tested using Shapiro–Wilk which indicated that data were non‐normally distributed for all outcome variables with the sole exception of the OCAI Clan subscale (*p* = .07). As a result, the non‐parametric Mann–Whitney *U* test was selected to compare pre ‐and post‐culture‐change programme intervention samples. To test whether repeaters formed a discrete group with distinct responses to the intervention, we used the Wilcoxon signed‐rank test to compare their pre‐ and post‐intervention scores. Open‐ended responses regarding the intervention were examined by two of the research team.

## 3 RESULTS

In total, *N* = 241 valid questionnaires were returned across the two data collection phases, *n* = 137 from the pre‐test and *n* = 104 from the post‐test iteration representing response rates of 46.1% and 35.0%, respectively. Listwise deletion at the outcome variable level meant that the final *n* for each outcome varied from 133 to 137 at baseline (Mdn = 136) and from 80 to 104 at Follow‐up (Mdn = 93). Sample characteristics at both points are detailed in Table 1. There were no statistically significant differences between the samples on the reported characteristics.

Comparisons revealed that non‐repeaters differed from repeaters at baseline on SAQ‐SF Teamwork Climate subscale (3.73 [0.60] versus. 4.08 [0.33], *U* = 687.5, *z* = −2.14, *p* = .32); SAQ‐SF Job Satisfaction subscale (3.23 [0.80] versus. 3.79 [0.42], *U* = 561.0, *z* = −2.98, *p* = .003); the OCAI Clan subscale (177.17 [74.54] versus. 235.44 [71.30], *U* = 536.50, *Z* = −2.731, *p* = .006); and on the OCAI Adhocracy subscale (187.94 [81.61] versus. 160.31 [54.73], *U* = 614.50, *Z* = −2.19, *p* = .03). There were no significant baseline differences between repeaters and non‐repeaters on measures of MET‐related educational need or physiological health emergency‐related knowledge.

Table 2 shows baseline and follow‐up mean item scores for each subscale of the SAQ‐SF and the 3‐item Safety Behaviour Scale. Significant change was only detected on the latter outcome where the trend was towards poorer safety behaviour. Table 3 and Figure 1 present OCAI subscale scores; the follow‐up sample rated the organisational climate to be significantly less clan‐like and significantly more adhocracy‐like in the post‐test period. As demonstrated in Tables 4 and 5, MET‐related educational needs did not alter across the study period and neither did physiological health care‐related knowledge as measured on the LITQ.

**Table 2:** Safety attitudes questionnaire mean item subscale scores at baseline and follow‐up

SAQ‐SF subscale	Baseline *M*(*SD*) *n* = 136	FU *M*(*SD*) *n* = 103	*U*	*Z*	*p*
Teamwork climate	3.78 (0.59)	3.74 (0.76)	6,980.5	−0.045	=0.96
Safety culture	3.85 (0.63)	3.89 (0.68)	6,739.5	−0.501	=0.62
Job satisfaction	3.30 (0.78)	3.19 (0.78)	6,291.0	−1.352	= 0.18
Stress recognition	3.86 (0.91)	3.95 (0.99)	6,456.0	−1.042	=0.30
Perception of unit management	3.81 (0.86)	3.78 (0.90)	6,915.0	−0.169	=0.87
Perception of hospital management	3.12 (0.97)	3.00 (0.90)	6,417.5	−1.11	=0.27
Working climate	3.33 (0.80)	3.23 (0.77)	6,432.0	−1.086	=0.28
Safety behaviour (elsous)	4.12 (0.72)	3.86 (0.70)	5,606.0	−2.679	**=0.007**

Bold indicates statistically significant results.

**Table 3:** OCAI total subscale scores at baseline and follow‐up

OCAI subscale	Baseline *M*(*SD*) *n* = 132	FU *M*(*SD*) *n* = 99	*U*	*Z*	*p*
Clan	184.23 (76.32)	158.77 (80.19)	5,307.5	−2.44	**=0.015**
Market	111.83 (51.17)	101.12 (45.31)	5,824.0	−1.41	=0.16
Adhocracy	117.09 (64.29)	150.86 (88.78)	4,744.5	−3.56	**<0.001**
Hierarchy	184.99 (79.20)	184.99 (68.68)	6,320.0	−0.43	=0.670

Bold indicates statistically significant results.

**Table 4:** MET‐related educational needs at baseline and follow‐up

Baseline *M*(*SD*) *n* = 133	Follow‐up *M*(*SD*) *n* = 95	*U*	*Z*	*p*
46.48 (5.03)	46.67 (5.69)	6,022.0	−0.65	=0.52

**Table 5:** LTIQ knowledge at baseline and follow‐up

Scenario	Baseline *M*(*SD*) *n* = 137 except * *n* = 136	Follow‐up *M*(*SD*) *n* = see footnote	*U*	*Z*	*p*
1	3.56 (2.03)	3.09 (2.31)^a^	5,618.5	−1.45	=0.15
2	2.27 (1.18)	2.07 (0.96)^b^	5,530.0	−1.51	=0.13
3	3.42 (1.76)	4.31 (10.46)^c^	5,896.5	−0.20	=0.84
4	1.54 (1.23)	1.29 (1.32)^d^	4,965.0	−1.63	=0.10
5	1.42 (1.51)	1.51 (1.38)^e^	5,061.5	−0.88	=0.38

^a^ *n* = 93.

^b^ *n* = 91.

^c^ *n* = 88.

^d^ *n* = 82.

^e^ *n* = 80.

Repeaters' scores significantly changed negatively over time on the SAQ‐SF Job Satisfaction subscale (3.79 [0.42] versus. 3.41 [0.51], *Z* = −2.16, *p* = .03) and SAQ‐SF Perception of Hospital Management (4.14 [0.44] versus. 3.86 [0.57], *Z* = −2.57, *p* = .01) (*n* = 17). Repeaters' scores on the OCAI subscales did not differ significantly between the two iterations (Adhocracy subscale *p* = .057). Repeaters scores did not differ significantly on the MET‐educational needs or LTIQ knowledge outcomes across time. Non‐repeaters scores did not differ between iterations on SAQ‐SF Job Satisfaction or SAQ‐SF Perception of Hospital Management subscale but did differ on measures of organisational culture to become less Clan‐like (177.17 [74.54] versus. 150.53 [78.66], *U* = 3,795.0, *Z* = −2.29, *p* = .02) and more adhocracy‐like (121.88 [65.21] versus. 157.40 [93.45], *U* = 3,402.0, *Z* = −3.29, *p* = .001).

Responses to an open‐ended questionnaire item about the study intervention did not generate sufficient data to draw conclusions about participants' views of it. Rather, this opportunity was more frequently used to express dissatisfaction with managerial culture.

## 4 DISCUSSION

The current study examined changes in objective scores of safety attitudes, organisational culture, health care‐related training needs and emergency care‐related knowledge among nurses working in inpatient mental health services across one health district in a metropolitan district of Sydney. The period between the two measurements included culture‐change interventions delivered by an organisational culture‐change expert to ward‐based nursing staff and to nurses in Service‐level executive leadership positions. Analysis has revealed that there were no measurable changes on safety attitudes, educational need or knowledge at follow‐up for the whole cohort. There was a small but statistically significant measured change in organisational culture away from a clan‐like culture and towards an adhocracy‐like culture. This finding cannot be immediately categorized as either positive or negative since the appropriateness of organisational characteristics for a particular working environment may differ across settings. We do know that a previous study (Dickens, Salamonson, et al., 2019) demonstrated that those who viewed organisational culture as market‐oriented had significantly poorer safety attitudes than those with other primary perceptions. As a result, we may conclude that, since the perceived market orientation was not changed, the measured difference on adhocracy and clan‐ness may be of little practical benefit. The lack of significant positive change is, of course, disappointing but certainly is not unusual for culture‐oriented change programmes conducted in mental health inpatient settings (e.g. Berg & Hallberg, 1999; Bjorkdahl et al., 2013). However, targeted leadership programmes have been linked with safety‐related culture among ‘frontline’ nursing staff in mental health settings (Kristensen et al., 2016), although interestingly in the same study there was more limited change among senior managerial staff.

Potential explanations for the failure of this essentially complex intervention to yield positive results on the outcomes measured may be explicable in multiple ways. One, of course, is that the intervention had no effect on participants' attitudes or subsequent practice in attitudinal or behavioural terms. However, this is only one of several possibilities given the matrix of potential interactions between the programme content (workplace *and* senior leadership elements), participants (shopfloor nurses and management), the inherent difficulties in ensuring that participants receive the right ‘dose’ of the intervention, the outcomes selected for measurement, and the disconnect between those outcomes and the interventions necessitated by the design in which participation in the intervention was mandated yet participation in the evaluation was not. Indeed, research (Machin & Treloar, 2004) has shown that motivation to learn when training is mandatory is largely predicted by perceived organisational commitment, that is, the level to which the individual feels attached to the organisation and the actions they take as a result (Meyer et al., 1993).

It is difficult to draw conclusions about subgroups of respondents based on the small proportion who provided information allowing their pre‐ and post‐intervention evaluations to be matched. However, analyses suggested that those who did do this started with relatively high perceptions of the SAQ‐SF perception of teamwork culture and job satisfaction subscales, and perceived a more clan‐like and less adhocracy‐type culture. At follow‐up, their scores on the SAQ‐SF job satisfaction and perception of hospital management subscales had declined. While this small subsample may not be representative, it is potentially informative that those with sufficient ‘buy‐in’ and commitment to the service to complete somewhat lengthy questionnaire batteries on two occasions commenced with some relative signs of optimism but actually fared more poorly over time on key SAQ‐SF measures. This begs the question as to whether interventions like the one trialled here can be assumed to be at least neutral and at best benign.

Also relevant are the results of a linked study conducted in the same service prior to the second iteration of data collection for the current study. Focus group interviews were used to explore mental health nurses' experiences and perceptions of providing care for severely deteriorating patients in mental health settings (Brunero et al., 2019). Analysis revealed themes linked to procedures and leadership accountability, confidence in the workforce and complexity of care and the environment. While some of the issues—notably within theme one—were related to organisational culture, it is certainly possible that most concerns were linked with resource availability, education and training. In this context, it seems clear that implementation of an organisational leadership programme in isolation from other developments will not maximize the potential for success.

### 4.1 Limitations

There was a relatively low response rate which was considerably poorer at follow‐up. We noted previously that the study questionnaires were time‐consuming and this may have deterred a second completion. The low number of participants who we were sure had completed both iterations provided evidence for this view. Some of the questionnaires (Elsous' SAQ‐SF Safety Behaviour addition, MET and LTIQ) lack external validity but we included since they were relevant to the study. The intervention was not specifically targeted at improving specific safety or knowledge outcomes, and it is therefore unsurprising that related outcomes did not change between measures. Given that the intervention was mandated and not subject to research consent, we have no relevant process data. Neither did we collect service‐level data on real‐world outcomes such as serious adverse events, although given their rarity in absolute terms it seems unlikely that any change could be attributed to anything other than natural variation over time. A review of adverse event reporting in randomized controlled trials has demonstrated that relevant events rarely reach levels where it is appropriate to statistically test them (Phillips et al., 2019). The culture‐change expert responsible for determining the content of and delivering the programme reported considerable difficulties in engaging those participating specifically in the leadership programme, and suboptimal support in helping shopfloor nurses to attend all three mandated sessions through the promised ‘backfill’ of positions. This was apparent in the attrition of nurses attending sessions two and, especially, three. Overall, full attendance at the workplace programme was only achieved by 30.6% of the nursing workforce. Further, we are unaware of any ongoing monitoring or cycle of audit and feedback implemented by the service management to gauge the effectiveness of the interventions, and changes arising from it. This is important since these approaches appear to be among the most effective in securing and sustaining change (e.g. Bloom, 2005).

## 5 CONCLUSIONS

We recognize the pressure on senior managers to be seen to be acting in accordance with recommendations arising from enquiries such as that which contextualized the current study. We also acknowledge the foresight shown in funding the current evaluation. The clear fact, however, is that the intervention failed to demonstrate any measurable value in terms of cultural, attitudinal or knowledge change. Inherent limitations of the study design exacerbate the issue because little weight can be put on judgements about the precise reasons behind the lack of intervention effect: Was it indeed without value or was it an artefact of inadequate measurement or outcome selection? As a result, we are in something of a double‐bind: we could not recommend a similar intervention in future yet we cannot confidently state that it has no value. Systematic reviews of research strongly suggest that organisational culture is linked to patient outcomes, though a lack of evidence from randomized controlled trials makes it difficult to disentangle causal associations (Braithwaite et al., 2017). Further, there is actually very little direct high‐level evidence that general culture‐change programmes effectively improve health care performance (Parmelli et al., 2011). While programmes like the one tested in this paper are based in evidence, they are essentially complex interventions according to criteria defined by Mills et al. (2019): (a) the extent to which the intervention comprises multiple interacting components; (b) the range, difficulty and variability of behaviours required by those delivering or receiving the intervention; (c) the extent to which multiple groups or organisational levels are targeted by the intervention; (d) the number and variability of outcomes measured; and (e) the degree of flexibility permitted in the intervention. As such, they require evaluation that may take longer to design, plan and implement than is often allowed by those seeking to be acting on recommendations.

## 6 IMPLICATIONS FOR NURSING MANAGEMENT

Findings demonstrate the need for service‐wide interventions aimed at cultural change to be carefully considered prior to deployment. Outcome measures should align as closely as possible to intended targets while recognizing that flexibility will need to be maintained in the intervention as it progresses. Managers need to recognize that organisational commitment of nurses is a prerequisite to culture development and that simply mandating attendance at an intervention is not a substitute for engagement and provision of a range of development activities that align with the concerns of frontline nursing staff.

## CONFLICT OF INTEREST

Michelle Taylor is principal consultant, Taylor Made Coaching Solutions, and was contracted by the local health district to provide the study intervention. The other authors have no conflicts of interest to declare.

## AUTHOR CONTRIBUTIONS

BE, YS, LR, MS and MT conceived and designed the study. AJ and KS acquired the data. GLD, BE and YS analysed and involved in interpretation of the data. GLD drafted the work. All authors revised the important intellectual content, made final approval of the manuscript and agreed to be accountable for all aspects of the work.

## ETHICAL APPROVAL

This study received ethical approval from South Western Sydney Local Health District Human Research Ethics Committee (HE17/198).
